# Effect of medical education on European psychiatrists’ knowledge in management of major depressive disorder and psychiatric emergencies

**DOI:** 10.1192/j.eurpsy.2022.316

**Published:** 2022-09-01

**Authors:** L. Thevathasan, L. Fairley, C. Phillips

**Affiliations:** Medscape LLC, Clinical Strategy, London, United Kingdom

**Keywords:** Suicide, major depressive disorder, Psychiatric emergencies, MDD

## Abstract

**Introduction:**

The challenge for psychiatrists is keeping up to date with the latest clinical trial data in managing major depressive disorder (MDD) and psychiatric emergencies.

**Objectives:**

We evaluated whether an online educational video lecture directed at psychiatrists, could improve knowledge and confidence regarding management of psychiatric emergencies associated with MDD.

**Methods:**

Educational effect was assessed using a 3-question repeated pairs, pre/post assessment survey. A paired-samples t-test was conducted to assess overall number correct and confidence change. A McNemar’s test was conducted to assess question-level significance. P values < 0.05 are statistically significant. Cohen’s d test was used to estimate the magnitude of effect of education. The activity launched on 8 April 2021, and preliminary data analysed as of 24 June 2021.

**Results:**

807 psychiatrists participated in the programme, of which 150 completed the pre- and post-assessment test. An average overall correct response rate of 44% pre- increased to 74% post- (67% relative increase, P<0.001; Cohen’s d = 0.91). Knowledge on the burden of suicide and MDD improved from 38% pre- to 85% post- (124% relative increase,P<0.001). Knowledge regarding clinical data for novel therapies for use in psychiatric emergencies improved from 47% pre- to 68% post- (45% relative increase, P<0.01). Knowledge regarding signs of suicidal intent in patients with MDD improved from 49% pre- to 71% (45% relative increase, P<0.001) following education.

**Conclusions:**

This study demonstrates the positive effect of online medical education on psychiatrists’ knowledge in contemporary management of psychiatric emergencies associated with MDD in Europe.

**Disclosure:**

The results of this study were from an educational programme that was developed through independent educational funding from Janssen Neuroscience.

